# Rotational strength, range of motion, and function in people with unaffected shoulders from various stages of life

**DOI:** 10.1186/1758-2555-1-4

**Published:** 2009-03-02

**Authors:** Jean-Sébastien Roy, Joy C MacDermid, Kirsty Usher Boyd, Kenneth J Faber, Darren Drosdowech, George S Athwal

**Affiliations:** 1 School of Rehabilitation Science, McMaster University, Hamilton, Ontario, L8S 1C7, Canada; 2 Hand and Upper Limb Centre, St. Joseph's Health Centre, London, Ontario, N6A 4L6, Canada

## Abstract

**Background:**

Different measurements are used to assess shoulder function, including range of motion, strength, functional performance and self-report function. To understand disablement, it is necessary to understand the relationship between impairments and function in persons without shoulder problems. This study was conducted to enhance existing comparative data in subjects without upper extremity pathology, and to assess the relationships between impairments (range of motion, strength) and self-reported or measured function/disability. The impact of age, gender and dominance was determined.

**Methods:**

Two-hundred ninety-four subjects with unaffected shoulders were recruited. The subjects (mean age: 37 years old) were divided into three subgroups, 18–39, 40–59, and over 60 years of age. During a single session, at least two of the following variables were measured: self-reported function (shoulder disability scales), range of motion, isometric rotational strength, or upper limb functional performance (FIT-HaNSA). Two-way analysis of variance was used to determine, for each variable, the effects of age and gender. The relationship between the outcomes was established using Pearson product correlations.

**Results:**

Men were significantly stronger than women for all age categories. There was an age-related decline in strength in men in the over-60 age category. Significant negative correlations between strength and range of motion were demonstrated (-0.22 <*r *< -0.32). Women had a significantly higher range of motion than men for external rotation in the 40–59 age category. Furthermore, the subjects in the over-60 age category experienced a decrease of range of motion. There was minimal disability reported in all age groups on self-report scales. Only the Simple Shoulder Test demonstrated significant decreases in the over-60 age category and correlated with age (*r *= -0.202).

**Conclusion:**

Self-reported disability was low in individuals without upper extremity problems, although recruitment of such individuals was difficult in the older age groups due to the high prevalence of shoulder pathology. A low correlation between self-report disability and strength/range of motion in these unaffected subjects reflects the lack of disability reported by all subjects without pathology despite normal variations in strength and motion.

## Background

There is an increased emphasis on using subjective and objective outcome measures to characterize function in persons with shoulder disorders [1-4]. In particular, outcome assessments increasingly encompass terminology and concept from the International Classification of Functioning, Disability and Health [5]. For shoulder disorders, impairments most commonly measured include range of motion and strength. Disability includes activity limitations and participation restrictions that are often best measured by self-report questionnaires. Activity limitations can also be assessed by performance-based tests. It is important to understand the relationship between impairments and measure of function/disability in persons without shoulder problems, as well as the effects of age and gender on these different aspects of shoulder function.

The importance of objective measurements of strength and range of motion when evaluating shoulders is evidenced by the frequency with which they are reported in the literature [2,4,6-9]. Objective assessment of strength provides information on integrity of the rotator cuff [10] and is used to gauge the recovery of muscle function following intervention [11,12]. Normative data is required for comparison to grade the extent of recovery. Otis *et al*. discussed the value of assessing strength relative to a matched population, especially in the presence of a shoulder deficit, in order to characterize these deficits [13]. Moreover, Hughes *et al*. noted the importance of having unaffected shoulders data for comparison in patients whose shoulder deficits manifest with bilateral involvement [14]. Kuhlman *et al*. acknowledged that while many studies have attempted to define normal values for the strength of shoulder muscles, there have been varying degrees of success because of a lack of standardization (plane of motion, position of the shoulder or stabilization of the body) [15].

Gender related differences in strength have been reported [14,16]. More specifically, Hughes *et al*. have shown that men are stronger than women when controlling for age and weight [14]. The effects of age and dominance, however, are less well known. It has been suggested that, in the normal population, age is negatively associated with isometric shoulder strength and that some shoulder rotational strength measurements differ between dominant and nondominant sides [14,16]. Studies examining range of motion and its relationships with age, gender and dominance, unfortunately, have reported varied results [17-19]. Most studies have reported that only some shoulder motions decrease with age [17,18], however, the specific shoulder ranges of motion affected by age are inconsistent between studies. As for gender-related effects, minimal differences between genders have been described by Murray *et al*. [18], while Barnes *et al*. observed greater range of motion in women as compared to men [17].

Measures of function provide a broader view of patient status and are considered more patient centered. Several studies have examined the contribution of self-report questionnaire to disability assessment, or reported on their validity [20,21]. Unfortunately, very few studies have established normative values for these self-report scales [21]. Furthermore, those that have presented normative values have not included a determination of how self-reported function is related to strength and range of motion [7,13-16,21,22].

Recently, an upper-extremity functional performance test, the FIT-HaNSA, has been proposed by MacDermid *et al *[23]. This standardized test was developed to assess functional performance for sustained upper extremity activity. The reliability and concurrent validity of the FIT-HaNSA have been established in persons with shoulder disorders. It has been shown to discriminate between persons with and without shoulder disorders. Normative data for the FIT-HaNSA has yet to be published. Furthermore, the relation between the FIT-HaNSA and strength, range of motion and self-reported function has not been established for individuals without shoulder pathology.

The purpose of this investigation is to enhance existing comparative data on normal shoulder range of motion and strength, and to add information on functional performance and self-reported function. A secondary purpose is to establish the inter-relationships between these different types of assessments and to evaluate the impact of dominance, age and gender. The hypotheses are that men will be stronger than women in all age categories. Furthermore, rotational strength, range of motion, functional performance and upper extremity function will be decreased in the over-60 age category compared to the 18–39 and 40–59 age categories.

## Methods

### Subjects

Two-hundred ninety-four subjects voluntarily participated in the study (121 men and 173 women). The subjects were divided into three age cohorts, 18–39, 40–59, and over-60. The mean subject age was 36.9 years and the range was from 18 to 79 years (Table 1). Subjects were first specifically recruited for this study by a variety of methods, including flyers advertising the study, people accompanying their spouses to the Hospital, and word of mouth (n = 128; variables evaluated: strength, range of motion and self-reported function). Separate groups of subjects participated in studies evaluating the psychometric qualities of the FIT-HaNSA (n = 125; variables evaluated: upper limb functional performance and self-reported function and/or range of motion and/or strength) and the concomitant validity of isometric strength devices (n = 41; variables evaluated: strength and self-reported function). Inclusion criteria were: 1) age 18 and above; 2) a self-reported normal upper limb function (no pain or movement limitation); 3) no history of surgery to the upper limb; and 4) no previous diagnosis or treatment to any part of the upper extremity. Subjects had to report that they had no problems with their shoulder at the time of the assessment. Most had no history of injury. Six subjects, however, had sustained fractures more than ten years previously (2 finger fractures, 2 radial head fractures, and two clavicle fractures). Two subjects also admitted to a remote history of lateral epicondylosis. All of these individuals characterized their injuries as distant, their recovery as complete and their upper limb function as normal and unaffected. Therefore, they were included. This study was approved by our Institutional Review Board.

**Table 1:** Subjects characteristics and self-report function in men and women by age.

	**n**	**Age (years)**	**Dominance**	**DASH**	**SST**	**WORC**
						
		Mean*	**Right/left**	Mean*	Mean*	Mean*
**Men**	121	39.2 ± 16.6	106/15	1.8 ± 3.1 (n = 100)	11.6 ± 1.6 (n = 60)	6.4 ± 12.0 (n = 59)
**18–39**	71	27.1 ± 5.5	62/9	1.8 ± 2.9 (n = 54)	11.9 ± 0.4 (n = 25)	5.8 ± 13.6 (n = 25)
**40–59**	30	48.5 ± 5.3	26/4	1.1 ± 2.9 (n = 27)	11.8 ± 0.5 (n = 19)	6.9 ± 12.7(n = 18)
**60+**	20	68.2 ± 5.7	18/2	2.6 ± 3.8 (n = 19)	10.9 ± 3.0 (n = 16)	6.9 ± 8.5 (n = 16)

**Women**	173	35.3 ± 15.1	157/16	1.8 ± 2.8 (n = 155)	11.7 ± 1.5 (n = 70)	5.4 ± 8.3 (n = 70)
**18–39**	112	25.4 ± 6.2	99/13	1.7 ± 2.4 (n = 96)	12.0 ± 0.2 (n = 27)	3.9 ± 3.9 (n = 27)
**40–59**	44	48.8 ± 5.1	42/2	2.0 ± 3.6 (n = 43)	11.8 ± 0.5 (n = 31)	7.7 ± 11.4 (n = 31)
**60+**	17	64.9 ± 4.8	16/1	1.9 ± 2.6 (n = 16)	10.9 ± 3.4 (n = 12)	3.1 ± 3.9 (n = 12)

**Total**	294	36.9 ± 15.9	263/31	1.8 ± 2.9 (n = 255)	11.7 ± 1.5 (n = 130)	5.9 ± 10.1 (n = 129)
**18–39**	183	26.1 ± 6.0	161/22	1.8 ± 2.6 (n = 150)	11.9 ± 0.3^a ^(n = 52)	4.8 ± 9.8 (n = 52)
**40–59**	74	48.7 ± 5.1	68/6	1.7 ± 3.4 (n = 70)	11.8 ± 0.5^b ^(n = 50)	7.4 ± 11.8 (n = 49)
**60+**	37	66.6 ± 5.5	34/3	2.3 ± 3.2 (n = 35)	10.9 ± 3.1^a, b ^(n = 28)	5.3 ± 7.1 (n = 28)

Significant differences for the SST between: ^a^18–39 and over-60 age categories; ^b^40–59 and over-60 age categories. * Mean ± 1 SD.

Abbreviation: n, Number of subjects.

The test session involved the measurement of at least two of the following variables: self-reported shoulder and upper extremity function, range of motion, strength or upper limb functional performance (FIT-HaNSA). Separate subgroups of volunteers were enrolled sequentially for the strength/motion and functional performance subsets for the study. The subjects were assessed by research assistants following standardized tests procedures.

### Test Procedures

#### Strength

Shoulder internal and external rotation isometric strength was measured in Newton metres (Nm) using a computerized dynamometer (LIDO WorkSET™; Loredan Biomedical, West Sacramento, California). These muscles groups were chosen since they are the ones usually involve in patients with shoulder disorders such as rotator cuff tendinosis or tear [8]. Testing was performed with subjects seated in a heavy straight-backed chair with both feet flat on the floor. The arm to be tested was positioned with 30° forward flexion and abducted to 45° (in the plane of the scapula) as per Leroux *et al*. [7]. The machine axis was adjusted to 45° so that it was aligned with the longitudinal axis of the humerus. The subject grasped a handgrip in neutral position for forearm pronation/supination. Isometric internal and external rotations were tested bilaterally with machine calibration between each test. Three measurements were averaged for each muscle group. The LIDO WorkSET™ has been shown to have excellent reliability in the measurement of isometric internal and external shoulder rotation strength with intraclass correlation coefficient (ICC) of 0.88 to 0.94 [8].

#### Range of Motion

Active and passive internal and external rotation range of motion was assessed bilaterally using a goniometer (in degrees). External rotation was measured in both the sitting and supine positions, whereas internal rotation was only measured supine. For sitting external rotation, subjects were seated in a straight-backed chair with both feet flat on the floor. Measurements were taken during active motion with the humerus at 0° of abduction and elbow at 90° of flexion. The olecranon process was used as the axis of rotation [20]. Supine external and internal rotations were measured passively with the humerus abducted in the frontal plane to 90° and the elbow flexed to 90°. The scapula was stabilized during internal rotation by the research assistant in order to avoid protraction of the shoulder girdle. The scapula was not stabilized in external rotation. Passive external/internal rotation of the shoulder was performed to subject tolerance with the olecranon serving as the axis of rotation as per the American Academy of Orthopaedic Surgeons [24]. The movement was stopped when the first resistance was felt. Intratester reliability of shoulder movements using a goniometer has been shown to be excellent with ICC of 0.88 to 0.93 [25].

#### Functional performance

The FIT-HaNSA was used to evaluate the upper-limb functional performance. The FIT-HaNSA is a test battery of 3 tasks that simulate activities of lifting and sustained overhead work [23]. In the first task, a shelf was placed at waist level and a second shelf was placed 25 cm above it. Three 1-kg containers were placed 10 cm apart on the lower shelf. Using the dominant arm, the patient had to lift the three containers, one at a time, from one shelf to the other at a speed of 60 beats per minute, controlled by a metronome. The subjects were instructed to do the test until five minutes have elapsed or they feel unable to continue. The time was measured in seconds by a stopwatch. In the second task, the shelves were adjusted so that one shelf was placed at the subject's eye level and the second shelf was placed 25 cm below it. The subjects had to lift the three containers between the shelves at a speed of 60 beats per minute. In the third task, a shelf was placed at the subject's eye level with an attachable plate, perpendicular to the shelf. Subjects had to use their dominant arm to repeatedly screw and unscrew bolts in a pattern into the plate. A 30 seconds rest period was given between each test. The same stopping protocol was used for the three tasks. The stopping criteria included subject fatigue and examiner's assessment of fatigue (substitute movement) [23]. A total score was calculated by averaging the time for the three tasks. The FIT-HaNSA has been shown to significantly discriminate between subjects with and without shoulder pathologies [23].

#### Self-report upper extremity function

Three self-reported disability scales were employed to assess shoulder and upper limb function. These included a joint-specific (Simple Shoulder Test [SST]), an upper-extremity disability (Disabilities of the Arm, Shoulder, and Hand [DASH]) and a condition-specific (Western Ontario Rotator Cuff [WORC]) questionnaire. All subjects completed at least one of these instruments. Each instrument varied with respect to the number of items included (SST with 12, DASH with 30, and WORC with 21) and with respect to time to completion (DASH and WORC in 3 minutes, SST in 1.5 minutes) [1]. The DASH measures physical disability and symptoms of the upper limb [26]. It addresses difficulty in performing various physical activities that require upper extremity function (21 items); symptoms of pain, activity-related pain, tingling, weakness and stiffness (5 items); or impact of disability and symptoms on social activities, work, sleep and psychological well-being (4 items). The DASH scores range from 0 (no disability) to 100 (most severe disability). The SST measures functional limitations of the affected shoulder [27]. The SST consists of dichotomous (yes [1] or no [0]) response options. For each question, the patients indicate that they are able or are not able to do the activity. The scores range from 0 (worst) to 12 (best). The WORC is a questionnaire developed to measure health related quality of life in patients with rotator cuff disease [28]. WORC consists of 21 items in 5 domains: physical symptoms (6 items), sports and recreation (4 items), work (4 items), lifestyle (4 items) and emotions (3 items). The WORC scores range from 0 (best possible) to 100 (worst possible). These three scales have been shown to be valid, reliable (test-retest reliability > 0.90) [28-30] and responsive (effect size/standardized response mean > 0.80)[29,31,32].

### Analyses

Descriptive statistics for strength, range of motion, functional performance and self-report for each age/gender cohort were calculated. Two-way analysis of variance (ANOVA) was used to determine, for each variable, the effects of age and gender. The factors in the model were gender (men or women) and age categories (18–39, 40–59, and over-60). ANOVA and independent t-tests, with Bonferroni adjustment, were used for multiple pairwise comparisons. Paired *t*-tests were used to evaluate the effect of dominance on range of motion and strength. To establish the inter-relationships between the different types of variables, correlations between age, strength, range of motion, functional performance and self-reported function were examined using Pearson product. The majority of the skewness and normality tests indicated normality was present. Some of the self-report data was skewed to low scores, but not sufficiently to warrant non-parametrics. An alpha level of 0.05 was used for all tests. All analyses were conducted with SPSS (12.0 for Windows, Chicago, Il, USA).

## Results

Descriptive statistics for strength, range of motion, self-report upper extremity function and upper-limb functional performance are included in Tables 1, 2, 3 and 4. The results are stratified by age category, gender, and hand dominance.

**Table 2:** Shoulder strength (in Nm) in men and women by age

	**Internal** **Rotation (D)**	**Internal** **Rotation (ND)**	**External Rotation (D)**	**External** **Rotation (ND)**
				
	Mean*	Mean*	Mean*	Mean*
**Men**	45.2 ± 15.8^¶ ^(n = 60)	42.8 ± 15.6^¶ ^(n = 60)	33.6 ± 11.3^¶ ^(n = 81)	34.0 ± 9.9^¶ ^(n = 60)
**18–39**	46.8 ± 16.5^¶ ^(n = 25)	45.7 ± 15.6^¶,a ^(n = 25)	33.6 ± 9.0^¶,a ^(n = 42)	35.8 ± 9.5^¶,a ^(n = 25)
**40–59**	50.5 ± 16.7^¶ ^(n = 18)	49.4 ± 15.5^¶,b ^(n = 18)	40.0 ± 11.8^¶,b ^(n = 21)	38.6 ± 8.6^¶,b ^(n = 18)
**60+**	37.1 ± 10.1^¶ ^(n = 17)	31.5 ± 8.8^¶,a, b ^(n = 17)	25.9 ± 11.2^¶,a, b ^(n = 18)	26.5 ± 7.7^¶,a, b ^(n = 17)

**Women**	20.7 ± 7.6^¶ ^(n = 71)	18.7 ± 6.6^¶ ^(n = 71)	17.0 ± 4.8^¶ ^(n = 88)	16.6 ± 5.2^¶ ^(n = 71)
**18–39**	23.2 ± 9.0^¶ ^(n = 28)	21.0 ± 7.2^¶ ^(n = 28)	17.2 ± 5.0^¶ ^(n = 43)	17.5 ± 5.7^¶ ^(n = 28)
**40–59**	18.8 ± 5.7^¶ ^(n = 31)	17.6 ± 5.6^¶ ^(n = 31)	17.5 ± 3.7^¶ ^(n = 32)	16.0 ± 2.9^¶ ^(n = 31)
**60+**	19.8 ± 7.1^¶ ^(n = 12)	16.2 ± 6.3^¶ ^(n = 12)	14.8 ± 5.9^¶ ^(n = 13)	15.8 ± 8.3^¶ ^(n = 12)

**Total**	31.9 ± 17.1^§ ^(n = 131)	29.7 ± 16.7^§ ^(n = 131)	24.9 ± 11.9^§ ^(n = 169)	24.6 ± 11.6^§ ^(n = 131)
**18–39**	34.3 ± 17.6 (n = 53)	32.6 ± 17.2 (n = 53)	25.3 ± 11.0 (n = 85)	26.2 ± 11.9 (n = 53)
**40–59**	30.5 ± 18.9 (n = 49)	29.3 ± 18.6 (n = 49)	26.4 ± 13.7 (n = 53)	24.3 ± 12.3 (n = 49)
**60+**	30.0 ± 12.4 (n = 29)	25.1 ± 10.9 (n = 29)	21.3 ± 10.8 (n = 31)	22.1 ± 9.5 (n = 29)

Significant differences between: ^¶^men and women; ^§^dominant and non-dominant sides; ^a^18–39 and over-60 age categories; ^b^40–59 and over-60 age categories. * Mean ± 1 SD

Abbreviations: D, Dominant arm; ND, Non-dominant arm.

**Table 3:** Range of motion (in degrees) in men and women by age.

	**Supine**				**Sitting**	
		
	**Internal Rotation** **(D)**	**Internal Rotation (ND)**	**External Rotation** **(D)**	**External Rotation (ND)**	**External Rotation** **(D)**	**External Rotation (ND)**
						
	Mean*	Mean*	Mean*	Mean*	Mean*	Mean*
**Men (n = 58)**	64.3 ± 16.0	67.1 ± 16.1	87.8 ± 10.1	85.1 ± 12.6	65.2 ± 15.0	64.0 ± 16.6^¶^
**18–39 (n = 25)**	61.5 ± 1 5.3	64.0 ± 13.4	90.7 ± 9.5	87.0 ± 13.1	66.2 ± 14.6	66.4 ± 16.4
**40–59 (n = 17)**	69.8 ± 14.3	72.8 ± 16.7	86.0 ± 9.0	84.3 ± 12.6	60.5 ± 15.4	58.7 ± 19.9^¶^
**60+ (n = 16)**	62.7 ± 18.1	66.0 ± 18.5	85.1 ± 11.3	83.1 ± 12.2	69.0 ± 14.9	65.9 ± 11.9

**Women (n = 72)**	70.1 ± 19.1	70.3 ± 18.5	89.5 ± 11.5	86.2 ± 13.3	69.3 ± 14.4	71.6 ± 17.5^¶^
**18–39 (n = 30)**	69.3 ± 17.2	67.7 ± 15.5	91.5 ± 12.4	87.3 ± 13.1	68.0 ± 13.4	70.9 ± 19.5
**40–59 (n = 31)**	73.9 ± 18.3	74.5 ± 18.1	89.9 ± 10.2	87. 2 ± 11.6	73.1 ± 14.9	73.9 ± 16.5^¶^
**60+ (n = 11)**	61.5 ± 24.7	65.2 ± 25.6	82.7 ± 10.5	80.5 ± 17.5	66.6 ± 17.9	67.2 ± 14.8

**Total (n = 130)**	67.5 ± 18.0	68.9 ± 17.5	88.7 ± 10.9^§^	85.7 ± 13.0^§^	67.7 ± 14.8	68.1 ± 17.5
**18–39 (n = 55)**	65.8 ± 16.7	66.0 ± 14.5	91.1 ± 11.1^a^	87.2 ± 13.0	67.3 ± 13.8	68.8 ± 18.1
**40–59 (n = 48)**	72.5 ± 17.0	73.9 ± 17.5	88.5 ± 9.9	86.1 ± 11.9	68.2 ± 16.2	68.3 ± 19.1
**60+ (n = 27)**	62.2 ± 20.6	65.7 ± 21.2	84.1 ± 10.9^a^	82.0 ± 14.3	68.0 ± 15.9	66.4 ± 12.9

Significant differences between: ^¶^men and women; ^§^dominant and non-dominant sides; ^a^18–39 and over-60 age categories; ^b^40–59 and over-60 age categories. * Mean ± 1 SD.

**Table 4:** FIT-HaNSA (in seconds) in men and women by age.

	**Task 1**		**Task 2**		**Task 3**	
			
	**Mean**	**SD**	**Mean**	**SD**	**Mean**	**SD**
**Men (n = 40)**	298.3	11.1	248.6	72.1	288.0	34.9
**18–39 (n = 29)**	300.0	0.0	249.3	63.9	292.1	29.3
**40–59 (n = 8)**	300.0	0.0	282.5	49.5	283.0	48.1
**60+ (n = 3)**	276.7	40.4	151.6	129.6	261.7	47.8

**Women (n = 85)**	294.9	21.8	239.7	82.1	282.7	44.5
**18–39 (n = 69)**	295.4	19.8	230.6	84.6	279.9	48.6
**40–59 (n = 13)**	290.9	32.8	274.0	62.8	293.8	15.8
**60+ (n = 3)**	300.0	0.0	300.0	0.0	300.0	0.0

**Total (n = 125)**	296.0	19.1	242.6^§^	78.8	284.4^§¶^	41.6
**18–39 (n = 98)**	296.8	16.7	236.2	79.2	283.5	44.0
**40–59 (n = 21)**	294.4	25.8	277.2	56.9	289.7	31.4
**60+ (n = 6)**	288.3	28.6	225.8	115.5	280.9	36.8

Significant differences compared to:^§ ^Task 1; ^¶^Task 2.

### Strength

Gender by age interaction effects were observed for both internal and external rotations strength (*F *> 3.50; *P *< 0.03; dominant and non-dominant sides). Therefore, the effects of both gender and age were evaluated separately. For all age categories, men were significantly (approximately 2 times) stronger than women (*P *< 0.001; Table 2). For men, there was a marked decrease in mean strength in the over-60 age category (Table 2). ANOVA revealed that this decrease was significant in the men over 60 years of age as compared to younger age group for external rotation strength, dominant and non-dominant sides, and for internal rotation strength, but only for the non-dominant side (*P *< 0.001) (Table 2). This effect was not observed in women. For all mean strength measurements, men were strongest in the 40–59 age category, while women were strongest in the 18–39 age category. Across all subjects, the dominant side was significantly stronger than the non-dominant side for internal (*t *= 3.142; *P *= 0.002) and external (*t *= 2.173; *P *= 0.032) rotation (Table 2). However, the mean differences between dominant/non-dominant sides were small and may not be clinically relevant (mean differences: 2.2 ± 8.0 Nm or 6.9% for internal rotation; 1.0 ± 5.2 Nm or 4.0% for external rotation). Across all subjects, the external/internal rotation strength ratio was 0.88 ± 0.39 for the dominant arm and 0.89 ± 0.25 for the non-dominant arm. For the dominant side, the ratio was significantly lower (*F *= 3.435; *P *= 0.035) in the over-60 age category (ratio = 0.76 ± 0.23) compared to the 40–59 age category (ratio = 0.99 ± 0.47) suggesting a relatively greater loss of external rotation strength in the older group. Gender and dominance had no significant effects on the external/internal rotation strength ratio. There were significant correlations between shoulder rotation strength (internal and external rotation, dominant and non-dominant sides) and external rotation range of motion in the sitting position (*r *ranges from -0.22 to -0.32; *P *< 0.05) (Table 5).

**Table 5:** Correlation between age, range of motion and strength.

			**Range of motion**					
			
			**Supine**				**Sitting**	
			
		**Age**	**Internal ** **Rotation** **(D)**	**Internal ** **Rotation** **(ND)**	**External ** **Rotation** **(D)**	**External ** **Rotation** **(ND)**	**External ** **Rotation** **(D)**	**External ** **Rotation** **(ND)**
**Age**		---	-0.05 (n = 130)	0.03 (n = 130)	-0.22* (n = 130)	-0.15 (n = 130)	-0.01 (n = 196)	-0.08 (n = 131)
**Strength**	**Internal ** **Rotation** **(D)**	-0.14 (n = 131)	-0.09 (n = 128)	-0.07 (n = 128)	-0.05 (n = 128)	-0.04 (n = 128)	-0.28** (n = 129)	-0.32** (n = 129)
	**Internal ** **Rotation** **(ND)**	-0.17 (n = 131)	-0.16 (n = 128)	-0.12 (n = 128)	-0.03 (n = 128)	0.01 (n = 128)	-0.23** (n = 129)	-0.27** (n = 129)
	**External ** **Rotation** **(D)**	-0.09 (n = 169)	-0.07 (n = 130)	-0.03 (n = 130)	-0.01 (n = 130)	0.04 (n = 130)	-0.22* (n = 131)	-0.28** (n = 131)
	**External ** **Rotation** **(ND)**	-0.16 (n = 131)	-0.13 (n = 128)	-0.09 (n = 128)	-0.05 (n = 128)	0.05 (n = 128)	-0.23* (n = 129)	-0.28** (n 129)

* Significant at *P *< 0.05; ** Significant at *P *< 0.01.

Abbreviations: D, Dominant arm; ND, Non-dominant arm

### Range of motion

There were no interaction effects between age and gender for range of motion in either supine or sitting positions. Gender effects were observed for seated external rotation range of motion for the non-dominant side (*F *= 4.725; *P *= 0.03), showing that women had significantly more range of motion than men for seated external rotation (*t *= 2.545; *P *= 0.012), especially in the 40–59 age category (*t *= 2.967; *P *= 0.006) (Table 3). Age effects were observed in the dominant side for external and internal rotation in the supine position (*F *> 4.000; *P *< 0.05). ANOVA reveals the effects were only significant in external rotation (*P *= 0.016), showing that across all subjects the over-60 age category experienced a significant decrease of range of motion as compared to the 18–39 age category (Table 3). Across all subjects, the dominant side had greater range of motion in external rotation in the supine position (*t *= 3.242; *P *= 0.002) as compared to the non-dominant side. Again, the differences between dominant/non-dominant sides were small (mean differences for supine external rotation: 3.0° ± 10.6° or 3.4%). There was significant negative correlation between age and external rotation range of motion (dominant side) in the supine position (*r *= -0.22, *P *< 0.011) (Table 5).

### Functional performance

For functional performance, no interaction, age or gender effects were observed. Age and gender did not predict scores of any of the three tasks or of the total score of the FIT-HaNSA. Across all subjects, the performance of task I (level reaching) was significantly better than on the tasks II (overhead reaching) and III (sustained overhead work), and task III was performed significantly better than task II (Table 4). There were significant correlations between the FIT-HaNSA total score and the DASH scores (*r *= -0.31, *P *< 0.001).

### Self-report upper extremity function

Self-report of shoulder and upper extremity function revealed no obvious trends, except for the SST for which age effects were observed (*F *= 4.492; *P *= 0.013). Therefore, age categories were compared across all subjects using ANOVA. The results show that SST scores were significantly decreased in the over-60 age category when compared to the 18–39 and 40–59 age categories (*P *< 0.011) (Table 1). There was also a significant correlation between age and SST scores (*r *= -0.20, *P *< 0.05) (Table 6). There were no significant correlations between self-report function and range of motion or strength. There was a significant correlation between the DASH and WORC (*r *= 0.19, *P *= 0.03). The SST was not correlated with the two others questionnaires.

**Table 6:** Correlation between self-reported function and age, range of motion and strength.

			**DASH**	**SST**	**WORC**
					
**Age**			0.03 (n = 255)	0.20* (n = 130)	0.05 (n = 129)
**Strength**	**Internal Rotation (D)**		-0.16 (n = 130)	0.06 (n = 130)	-0.05 (n = 129)
	**Internal Rotation (ND)**		-0.13 (n = 130)	0.07 (n = 130)	-0.02 (n = 129)
	**External Rotation (D)**		-0.12 (n = 130)	0.11 (n = 130)	0.07 (n = 129)
	**External Rotation (ND)**		-0.11 (n = 130)	0.15 (n = 130)	0.00 (n = 129)
**ROM**	**Supine**	**Int. Rot. (D)**	0.02 (n = 127)	0.03 (n = 127)	-0.06 (n = 126)
		**Int. Rot. (ND)**	-0.03 (n = 127)	0.00 (n = 127)	-0.03 (n = 126)
		**Ext. Rot. (D)**	-0.01 (n = 127)	0.02 (n = 127)	-0.13 (n = 126)
		**Ext. Rot. (ND)**	-0.02 (n = 127)	-0.08 (n = 127)	-0.02 (n = 126)
	**Sitting**	**Ext. Rot. (D)**	0.12 (n = 193)	0.16 (n = 128)	-0.10 (n = 127)
		**Ext. Rot. (ND)**	0.13 (n = 128)	0.03 (n = 128)	-0.02 (n = 127)

* Significant at *P *< 0.05.

Abbreviations: D, Dominant arm; ND, Non-dominant arm; ROM, Range of Motion; Int.

Rot., Internal Rotation; Ext. Rot., External Rotation.

## Discussion

This study provides comparative data for strength, range of motion, functional performance and self-reported function in persons who state they have no shoulder pain or disorders. The data is sparse for the over-60 group since it was difficult to find people in this age group who could fit the criteria. This reflects the high prevalence of shoulder problems in advancing age groups [33-35]. Random sampling to provide normative data from the population would be expected to include a high rate of people with shoulder pathology [35], therefore, the difference between unaffected and normative data should widen as the age of interest increases.

Results for shoulder rotation strength were mostly consistent with the findings of several previous papers that tested isometric strength, showing that on average, men are stronger than women [11,13-15]. Some differences, however, were observed. In men, strength significantly decreased in the over-60 age category. This differs slightly from the findings of Hughes *et al*. who suggest that strength declines linearly with age [14]. One possible cause of the decline in strength with aging is a decreased muscle mass, which may affect men (who have typically more muscle mass) more than women. Our data shows that there is a drop-off in strength around 60 years of age. Since retirement age is generally between 60 and 65, this might suggest that activity level is a contributing cause. Women appear to be strongest in their childbearing years (ages 18–39), which may be related to the type and quantity of activities required at this portion of their lifespan. Kim et al. [35] also found less age related decline in women as compared to men when evaluating four subgroups aged 40 years and older. It is clear that there is wide variation in strength in the younger categories, and that this variation decreases considerably in the eldest category; for the 18–39 category, the standard deviation for strength measurements ranges from 11.0 to 17.9, while in the over-60 category, the range is only from 9.5 to 12.4. This reduced variability may reflect a reduced contribution of occupation related inter-individual differences in the over-60 group.

Kim et al. [35] noted that 20% of subjects aged between 60–69 years, and 41% of subjects older than 70 years, claiming to be asymptomatic had a rotator cuff tear on ultrasonography. Although the numbers in the study subgroupps were small, the authors reported no significant loss of external rotation or abduction strength in partial or full-thickness rotator cuff tear; whereas a loss of abduction strength was observed with a large-to-massive full thickness tear. We did not perform imaging to define cuff status but recruited individuals reporting no shoulder pain or disability.

Muscle bulk may contribute to differences in range of motion between men and women. There were moderate negative correlations (-0.28 to -0.32) between strength and sitting range of motion. Due to testing position, muscle bulk may limit flexibility in stronger individuals. This interference would be eliminated in the supine testing due to the abduction of the humerus to 90°. Overall, there was a reduction in external rotation range of motion in the over-60 age category, while there was a negative correlation between age and external rotation range of motion. Our results are consistent with other studies that have shown that some shoulder range of motion decreases with aging [17,18]. However, in the study performed by Barnes *et al*., passive and active internal rotation at 90° of abduction was not affected by aging [17].

It has been suggested that isometric strength testing may provide more useful data in older patients with rotator cuff repairs than isokinetic tests [36]. Therefore, we elected to only perform isometric testing. Only few studies have reported normative data for external and internal rotational strength in the plane of the scapula [14,15]. This position has been described by Greenfield *et al*. as being clinically significant because it optimizes the length-tension relationship of the shoulder abductors and rotators and creates a significant degree of conformity between the humeral head and the glenoid [37]. Position can affect strength scores obtained so data comparisons should be made across similar test procedures.

The findings on the relationships between self-report scales in this study are counter-intuitive and incongruent with the results obtained in patient samples. One would expect there to be strong correlations between the self-report instruments because they all measure similar constructs and because strong correlations have been reported across different shoulder pathologies (0.88 <*r *< 0.91) [20]. Weak correlations, however, were observed (0.01 <*r *< 0.19). This may be due to attribute and scale factors. Firstly, the self-report scales were not designed to detect differences across a range of "normal" ability. Since a small range of the spectrum (minimal disability) was sampled in asymptomatic subjects, correlations obtained would not accurately portray the relationship across the full spectrum. Similarly, whereas a moderate relationship has been observed between self-reported function and measured of physical impairments (strength or range of motion) in studies assessing pathological groups, we found a weak relationship (0.18 < r < 0.56) [20]. This suggests that there is a wide variation in strength and range of motion that allows normal people to perform their normal tasks of daily life.

Unlike the DASH or WORC, a significant correlation between age and the SST was observed (r = -0.202). The SST contains a wider range of items, including more difficult items like questions on activities requiring muscle strength and power, such as lifting 3.6 kg (8 pounds), carrying 9.1 kg (20 pounds) or tossing a softball over-hand 18.3 m (20 yards). It also requires a yes/no response which may be more discriminative. The DASH asks about some higher level activities in a more indirect way saying "recreational activities which you take some force or impact through your arm, shoulder or hand". Perceptions or expectations around what this means to an individual may change overtime (response shift). Furthermore, most items on the DASH are less demanding activities of daily life tasks and the response is a 5-point scale so that changes in capability may be measured differently than on the SST. Similarly, the WORC also contains few specific difficult tasks and it measures on a 10-cm VAS scale. Thus, the weak relationships between the SST and the DASH or WORC may reflect their different approaches to measuring shoulder function.

The FIT-HaNSA is an alternative means to assess functional status that evaluates task performed in a timed test [23]. The FIT-HaNSA requires that subjects possess enough shoulder muscle endurance and joint range of motion to perform three different types of task. As with self-reported function, no significant correlations were observed between strength/range of motion and functional performance. This may be related to the spectrum issue. However, the FIT-HaNSA contains tasks more related to muscle endurance than to muscle strength. Therefore, future studies should look at the relationships between muscle endurance and the FIT-HaNSA. It is not surprising that no correlation with rotational range of motion and FIT-HaNSA were established as the test requires most of the movement in the frontal plane. The ranges of motion observed in the subjects recruited (the minimum was 32° in internal and 38° in external rotation) were more than enough to enable the subjects to perform the three tasks of the FIT-HaNSA. Unfortunately, shoulder elevation (flexion, scaption or abduction) ranges of motion were not evaluated in this study, but might be expected to have a bigger impact on the FIT-HaNSA scores.

The mean scores on the DASH index in this study fall below the normative values quoted in the study published by Hunsaker *et al*. [21]. For example, DASH normal scores were 10.1 ± 14.7 in the study by Hunsaker *et al*. [21], and 1.8 ± 2.9 in the present study. This suggests that we were successful in obtaining subjects with no upper extremity pathology for our study, and confirms our assumption that "normative" data is influenced by the high rates of shoulder pathology at older ages. This is important when considering "recovery" for older persons who previously were unaffected by shoulder pathology. While their scores may end up close to population norms, this might still represent a substantial loss from their preinjury level. We were easily able to recruit younger age cohorts, and there were large numbers of older people who were willing to be tested but did not qualify for the study criteria. In fact, the smaller size of this subgroup is a limitation in this study and suggests that future research on pain free shoulder in the elderly will be a challenge. Thus, we called our data unaffected comparison data, not normative data, since it reflects a non-pathologic state that is not "normal" in older age groups. The small number of older subjects could have also been detrimental in detecting differences across age categories.

## Conclusion

This paper yields several important facts about the relationship among rotational strength, range of motion, functional performance and self-reported function in people with unaffected shoulders and can serve as a valuable clinical resource for comparison with a patient population. First, there is indeed a decline in strength with aging, especially for the men. This decline, however, does not appear linear, but is more notable over the age of 60. There is more variation in strength within younger age subgroups than within the older subgroups. Second, there is a significant negative correlation between strength and sitting range of motion. In the sitting position, women are more flexible than men in external rotation for the 40–59 age categories. Third, there is no significant relationship between self-reported function and strength/range of motion over a narrow range of normal function in asymptomatic individuals. Finally, measured functional performances also not correlated with strength/range of motion in asymptomatic individuals; however, it is correlated with self-reported function (DASH). The SST may be more discriminative to age related changes in shoulder function than the DASH or WORC. Further research is needed to expand upon the interactions delineated in this paper, and to further understand the relationship between different impairment and disability outcome measures.

This study emphasizes that both young and older individuals who present as asymptomatic experience very low levels of disability; but the older individuals scores are much better than population "norms". Selection of comparative data should consider that scores from patients with similar procedures/injuries, or individuals without pathology are different, both conceptually and quantitatively, from a "normal" score which reflects the overall rate of functional disability existing in a population or subgroup.

## Abbreviations

ANOVA: Analysis of Variance; DASH: Disabilities of the Arm, Shoulder, and Hand; FIT-HaNSA: Functional performance of the upper extremity and neck; ICC: Intraclass Correlation Coefficient; Nm: Newton metre; SST: Simple Shoulder Test; WORC: Western Ontario Rotator Cuff index.

## Competing interests

The authors declare that they have no competing interests.

## Authors' contributions

JSR participated in the analysis and the interpretation of data, conducted statistical analyses, and drafted the manuscript. JCM: designed the study, participated in the analysis and the interpretation of data, and drafted the manuscript. KUB enrolled and tested subjects, and participated in the revision of the manuscript. KF participated in the development of the study question, enrolled subjects, and participated in the revision of the manuscript. DD participated in the development of the study question, enrolled subjects, and participated in the revision of the manuscript. GA participated in the development of the study question, enrolled subjects, and participated in the revision of the manuscript. All authors read and approved the final manuscript.
